# Genetic and Ontogenetic Variation in an Endangered Tree Structures Dependent Arthropod and Fungal Communities

**DOI:** 10.1371/journal.pone.0114132

**Published:** 2014-12-03

**Authors:** Benjamin J. Gosney, Julianne M. O′Reilly-Wapstra, Lynne G. Forster, Robert C. Barbour, Glenn R. Iason, Brad M. Potts

**Affiliations:** 1 School of Plant Science, University of Tasmania, Hobart, Tasmania, Australia; 2 School of Agricultural Science, University of Tasmania, Hobart, Tasmania, Australia; 3 National Center of Future Forest Industries (NCFFI), University of Tasmania, Hobart, Tasmania, Australia; 4 The James Hutton Institute, Craigibuckler, Aberdeen, Scotland, United Kingdom; Henan Agricultural University, China

## Abstract

Plant genetic and ontogenetic variation can significantly impact dependent fungal and arthropod communities. However, little is known of the relative importance of these extended genetic and ontogenetic effects within a species. Using a common garden trial, we compared the dependent arthropod and fungal community on 222 progeny from two highly differentiated populations of the endangered heteroblastic tree species, *Eucalyptus morrisbyi*. We assessed arthropod and fungal communities on both juvenile and adult foliage. The community variation was related to previous levels of marsupial browsing, as well as the variation in the physicochemical properties of leaves using near-infrared spectroscopy. We found highly significant differences in community composition, abundance and diversity parameters between eucalypt source populations in the common garden, and these were comparable to differences between the distinctive juvenile and adult foliage. The physicochemical properties assessed accounted for a significant percentage of the community variation but did not explain fully the community differences between populations and foliage types. Similarly, while differences in population susceptibility to a major marsupial herbivore may result in diffuse genetic effects on the dependent community, this still did not account for the large genetic-based differences in dependent communities between populations. Our results emphasize the importance of maintaining the populations of this rare species as separate management units, as not only are the populations highly genetically structured, this variation may alter the trajectory of biotic colonization of conservation plantings.

## Introduction

Plant genetic variation has an extended effect on dependent community and ecosystem processes in many differing systems [1]. Foundation tree species have been a focus of research due to their disproportionate influence on the surrounding environment [2]. Trees can act as community and ecosystem drivers, creating locally stable environmental conditions for communities to develop and ecosystem processes to operate, making them important in conservation and restoration research [3], [4]. Conservation and restoration research has mainly concerned ecological issues, such as which species and what type of species mixes to include and what kind of regeneration planning to implement. Less research has focused on the importance of genetic factors in forest restoration [5]. The influence of genetic variation across the range of a species has become an important issue for conservation and restoration planning, particularly as there is increasing interest in species and provenance translocations to account for climate change [6]. While most work has focused on adaptive impacts of translocation, little is known of the possible effects of such translocations on dependent communities [7].

The effects of genetic variation on foundation trees can occur at multiple genetic scales. Variation in extended genetic effects on dependent communities can occur across geographic races [8], populations within races, families within populations and clones [2]. Strong extended genetic effects have been well documented in the North American *Populus* system, which established a framework for community and ecosystem genetics research using clonally replicated tree genotypes planted in common gardens [2]. Recent literature has raised other factors, such as environmental variation, as being important in community composition, which may outweigh genetic-based effects [9], [10]. Another factor that has rarely been examined is developmental change.

In addition to genetic variation, developmental changes in plants are known to affect herbivory and physicochemical processes [11]. Most plants are under some form of genetically controlled developmental change (ontogenetic variation) in phenotypic traits [12]. Such ontogenetic variation leads to changes in gene expression through development [13] and can result in dramatic changes in morphology through an individual's life stages [12]. Heteroblasty is one such case whereby the leaves of a plant can change markedly from juvenile to adult ontogenetic stages [12]. Heteroblasty is no better illustrated than in the genus *Eucalyptus*, where significant differences in morphology and physicochemical properties occur between juvenile and adult foliage of many species [14].

The tree genus *Eucalyptus* dominates the landscape of Australia, comprising nearly 900 taxa (EUCLID 2006). A single tree can support a large abundance and diversity of organisms, influencing ecological processes [15]. While there have been numerous studies of tree genetic effects on single organisms in many eucalypt species [16], [17], extended effects of intra-specific genetic variation on dependent communities [8] and ecosystem processes [18] have only been studied in one widespread species, *Eucalyptus globulus*, due to its commercial importance in the plantation industry. However, the genus *Eucalyptus* has many rare species, often occurring naturally as small isolated populations which can be highly genetically differentiated [19]. Understanding how communities respond to genetic variation in rare species will allow for improved management of the species and their dependent communities [3], [20]. This study examines the dependent arthropod and fungal community of the heteroblastic tree species *Eucalyptus morrisbyi*, a rare eucalypt species restricted to just four small populations in Tasmania. This species is of particular relevance due to the ongoing activity in *in situ* and *ex situ* conservation [21].

In this study, we examined the genetic and ontogenetic variability of *E. morrisbyi* in relation to dependent arthropod and fungal communities. We assessed the two main populations of *E. morrisbyi* in a single common environment trial, addressing four main questions: First, does genetic and ontogenetic variation influence community composition? Second, what is the relative influence of these genetic and ontogenetic effects? Third, what are the key organisms responsible for community responses? Finally, what are the mechanisms driving differences in communities and what is the impact of previous marsupial browsing?

## Materials and Methods

### Ethics Statement

The sampling performed in this study of the rare species *E. morrisbyi* was undertaken under a Tasmanian Department of Primary Industries, Parks, Water and Environment permit (TLF14060). Permission to access the trial was obtained from Forestry Tasmania.

### Study Species

*Eucalyptus morrisbyi* is endemic to the island of Tasmania, Australia. The species consists of four populations, two main populations and the other two with only a few individuals [21]. The two main populations of the species, Risdon Hills (42°49′S, 172°20′E; 81 mature trees; RH) and Calverts Hill (42°56′S, 147°31′E; 1915 mature trees; CH), are separated by nearly 20 km and exhibit high molecular genetic variability both between and within populations [21]. As part of conservation efforts for the species, *ex situ* plantings at multiple sites capture a large array of the genetic diversity in both of the main populations. These plantings have recently revealed genetic-based variation in *Trichosurus vulpecula* (brushtail possum) browsing with the CH population being heavily browsed, causing poor growth and delayed ontogenetic transition to the adult foliage [22]. This genetic-based resistance is related to key physicochemical traits differing between the populations [22].

### Common Environment Trial

The common garden field trial used for the study consisted of families from the two main native populations of *E. morrisbyi*, Calvert's Hill and Risdon Hill. It was established at Geeveston, Tasmania, Australia (43°09′S, 146°51′E) in October 1999 from seedlings grown from open-pollinated seed from maternal trees in the natural populations. Seed from each maternal tree is a family and 30 families from each of the CH and RH populations were planted in a randomized block design [22]. A total of 480 trees were planted in eight replicated blocks of alternating rows of CH and RH individuals, with each family represented as a single-tree plot within each replicate. We assessed population and family level variation in the dependent arthropod and fungal community and physicochemical properties of leaves from 222 progeny (129 CH; 93 RH) in this trial with juvenile foliage. While most of the CH trees were in the juvenile leaf stage seventy-nine of the individuals from the RH population displayed both juvenile and adult foliage, allowing for assessment of the effect of ontogenetic variation (ie. different foliage types) within this population.

### Community Assessment

Trees were sampled on May 22^nd^ and 23^rd^ of 2007. Samples for community assessment were collected as single branches cut from the mid canopy of the northeast aspect of individual trees. Leaves were removed from the branches in the laboratory, total dry weight obtained, and number of leaves estimated. Following previous approaches [8], [23], dependent organisms were identified based on the presence of their causal symptom on damaged leaves. This technique is widely used in herbivore and community studies [24]–[26] as an alternative to live organism assessment. While it is a useful technique for such large studies, this approach does have conceptual limitations. For example, symptom abundance and richness may be unrelated to actual organism abundance and richness [27]. A single identified symptom may be caused by many individuals or may be covering previous damage by an unidentified organism. In contrast, a recent study has shown that leaf-chewer symptoms can be used to interpret insect richness and composition as they were found to be correlated in both the fossil record and living forests [28]. In this study, organisms identifications based on damage type were classified to species level where possible and genus level where not, based on publications and previous field and lab observations (see Table S1 and Figure S1). A few damage types were classified as unknown due to lack of observable evidence of causal organisms performing the damage. The raw abundance scores for putative causal organisms used in the analysis of this study represent the percentage of leaves affected by a given symptom from a ten-gram sample of leaves.

### Assessment of Physicochemical Properties of Leaves

Samples for assessment of the physicochemical properties of leaves were collected in the same manner as the community samples, with single branches cut from the mid canopy of the northeast aspect of individual trees. Ten to thirty fully expanded leaves from the sampling years growth were collected from each branch, freeze-dried and analyzed for their physicochemical differences using near-infrared spectroscopy (NIR). Freeze-dried leaves from each sample were scanned using a Bruker MPA FT-NIR spectrometer with a fiber-optic probe. Spectral wavelengths for each sample were obtained from the 780–2500 nm range at a 4 cm^−1^ resolution. Five leaves per sample were scanned four times on either side of the midrib toward the tip and base of each leaf, resulting in forty scans per sample. Recorded wavelengths for each sample are the means of all scans from the five leaves (8 scans per leaf; 40 scans total). The 220 wavelengths obtained from the NIR analysis were reduced to 20 principal components, which were used to summarize the variation in physicochemical properties [29], these will be referred to as NIR PCs from here on in. Prior to conversion of wavelengths, the NIR spectra was narrowed to 1300–2300 nm due to minimal variation in the spectra from 780–1300 nm and underwent standard smoothing procedures using 11 smoothing points with Unscrambler (Version 9.6; CAMO ASA, Oslo, Norway).

### Trichosurus vulpecula Browsing Assessment

Mann et. al. (2012) showed clear genetic-based differences in *T. vulpecula* browsing susceptibility of *E. morrisbyi* trees assessed in the same experimental common environment trial used in this study. Variation in *T. vulpecula* damage was linked to differences in key chemical and physical foliar properties. Due to the degree of impact that this marsupial herbivore has on the trees, it is possible they influence the dependent arthropod and fungal community. Hence, in this paper we examine the relative influence that previous browsing by *T. vulpecula* has had on the dependent community. Assessment of *T. vulpecula* browsing on each tree across the trial was performed in 2005 [22]. Damage scores were visually estimated for each tree on a five point scale based on their percent damage to total leaf area: 0 =  no damage, 1 = 1–25%, 2 = 26–50%, 3 = 51–75% and 4 =  greater than 75%.

### Multivariate Community Analysis

The influence of genetic and ontogenetic variation on dependent arthropod and fungal community composition were tested separately in Primer (version 6.1.3; Roborough, Plymouth, UK) using two models (model I & II; Table 1), with population and foliage type treated as fixed effects and replicate (spatial variation within the trial) and family within populations treated as random effects. The weighted community data were standardized to the unit maxima for each symptom in order to reduce disproportionate effects of highly abundant symptoms [30]. A Bray-Curtis dissimilarity matrix based on the standardized symptom data was used to represent community composition dissimilarity amongst samples. The significance of genetic and ontogenetic variation was tested using a permutational multivariate analysis of variance (PERMANOVA – Anderson 2008) of the dependent arthropod and fungal community within the Bray-Curtis dissimilarity matrix. A canonical analysis of principal components (CAP) [31] of the community Bray-Curtis dissimilarity matrix of all juvenile and adult samples, maximizing group differences, was performed to provide a visual interpretation of the community variation between both populations and foliage types.

**Table 1:** Mixed-models used in the study for analysis of significance and the partitioning of effect sizes.

Analysis model	Model description
Model I	y = μ+ *Replicate* + **Population** + *Family(Population)* + *Residuals*
Model II	y = μ+ *Replicate* + **Foliage Type** + *Residuals*
Model III	y = μ+ *Replicate* + NIR PCs + **Population** + *Family(Population)* + *Residuals*
Model IV	y = μ+ *Replicate* + NIR PCs + **Foliage Type** + *Residuals*
Model V	y = μ+ *Replicate* + Browsing + NIR PCs + **Population** + *Family(Population)* + *Residuals*
Model VI	y = μ+ *Replicate* + NIR PCs + Browsing + **Population** + *Family(Population)* + *Residuals*
Model VII	y = μ+ *Replicate* + NIR PCs + **Population** + *Family(Population)* + Browsing + *Residuals*

Fixed effects for each model are in boldface, random effects and residuals are italicized and covariates are underlined. Ordering of effects and covariates in each model are those used in the Type I SS analyses described in the methods.

The relative influence of physicochemical properties of leaves on the dependent community associated with genetic and ontogenetic variation were tested by including the twenty NIR PCs as covariates in their respective PERMANOVA analyses with sums of squares (SS) calculated sequentially (Type I SS). Spatial variation within the trial (replicate) was accounted for first in all analyses in order to remove the effect before the main effects of interest using models III and IV (Table 1). Similarly, a test for the influence of *T. vulpecula* browsing was conducted, but only for the genetic model, by including browsing scores with physicochemical properties of leaves as covariates. Three separate analyses of these influences on the dependent community were performed using models V, VI and VII (Table 1).

The proportion of variation influencing dependent community composition was calculated using components of variation obtained from the PERMANOVA analysis for each effect and covariate, following Anderson, et al. (2008). Total variance in each model was calculated by adding these components of variation and the residual variance, with negative components treated as zero [32]. The twenty components of variation obtained for physicochemical properties of leaves were compiled by summing all twenty components to represent their holistic influence on the dependent community. The percent of genetic and ontogenetic variation in dependent community composition accounted for by physicochemical properties of leaves was calculated from the components of variation obtained from the analyses with NIR PCs included and excluded (model I vs. III and model II vs. IV). This was also done to determine the percent variation attributed to genetic variation and physicochemical properties in dependent communities accounted for by *T. vulpecula* browsing (model III vs. V).

### Community Parameters Analysis

Community parameters were calculated for each sample from the weighted symptom scores using *BiodiversityR* in R. The parameters calculated included richness, abundance, Shannon diversity index and Pielou's evenness. Community parameters were analysed for genetic variation using a mixed model (model I) with the significance of random effects tested with the one-tailed likelihood ratio test and the fixed population effect with a Wald-F test. A paired sample t-test was used to analyse variation in community parameters between foliage types. Significance values for population effects and foliage types effects were adjusted to p = 0.0125 for community parameters using the Bonferroni method to correct for false discovery rates due to multiple testing [33]. Analyses were performed using *asreml* and *t.test* in R 2.15.3 [34].

### Analysis of Individual Symptoms

Individual symptoms were analyzed using a nonparametric Kruskal-Wallis test for population differences and a paired sample t-test for foliage type differences on the standardized abundance scores. Only symptoms found on at least ten percent of all trees from their respective data sets were subject to univariate analyses. Significance values were corrected using Bonferroni adjustment of p-values based on the number of organisms analyzed from each data set to account for multiple testing. Symptoms found significant in the univariate analyses were then used to determine which symptoms were influencing the variation in community composition between populations and foliage types. Univariate analyses of symptoms were undertaken using *kruskal.test* and *t.test* in R 2.15.3.

Significant symptoms were ranked based on their univariate Kruskal Χ^2^ or t-test values, from highest to lowest (1 =  highest, 2 =  lowest). Following the principles of stepwise multiple regression [35], these symptoms were sequentially excluded from calculations of the Bray-Curtis dissimilarity matrices used in multivariate PERMANOVA community analyses (models V and IV) until the significance of population and foliage type effects were lost in their respective analyses. Following the loss of significance of the effect of interest, the symptoms removed were individually included back into the PERMANOVA community analyses to validate their significance. The final resulting exclusions were those found to be the main contributors to variation in community composition between populations and foliage types.

## Results

### Genetic Variation in Foliar Organism Responses

**Figure 1:**
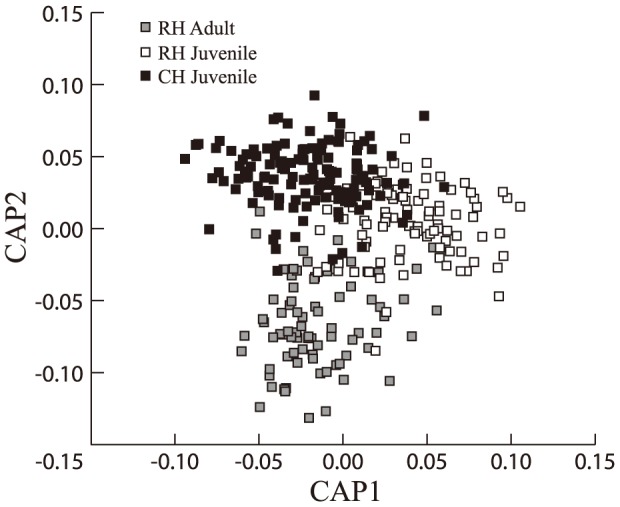
Canonical plot summarizing the variation in foliar community composition between populations and ontogenetic stages of *E. morrisbyi*. The constrained canonical analysis was undertaken on the principal coordinates derived from the Bray-Curtis dissimilarity matrix of all samples with the three a priori groups indicated summarizing the multivariate community variation between *E. morrisbyi* populations and foliage types. Each point represents an individual sample within the common garden trial. Pairwise Permanova+ analysis showed significant variation between all three groups (CH juvenile – RH juvenile, t = 3.6, p = <0.001; RH juvenile – RH adult, t = 3.6, p = <0.001; CH juvenile – RH adult, t = 5.0, p = <0.001). RH adults are grey squares, RH juveniles are white squares, and CH juveniles are black squares.

A total of 60 symptoms of arthropod and fungal species were identified on the sampled juvenile leaves between the two populations in the common garden trial. We found highly significant differences in arthropod and fungal communities on the juvenile leaves from RH and CH populations (PERMANOVA model I, Pseudo-F_1,221_ = 10.9, p<0.001), however the family within population effect was not significant (PERMANOVA, Pseudo-F_53,221_ = 0.99, p = 0.557). This significant variation is also shown in the ordination space derived from the CAP analysis with the two populations clearly separated (Figure 1). Spatial variation within the trial also significantly affected community composition (PERMANOVA, Replicate: Pseudo- F_5,221_ = 1.43, p = 0.005), however, components of variation obtained from the PERMANOVA analysis showed that its influence was small (1.2% of total variation) when compared with the population (genetic) effect (9.5%). To test if the difference between populations was due to an effect of the presence of adult foliage on the RH population, we tested for a difference in community composition between RH trees with only juvenile foliage and those with both juvenile and adult foliage. This difference was not significant (PERMANOVA, Pseudo-F_1,92_ = 1.15, p = 0.28), indicating the population differences were not due to the presence of adult foliage.

**Figure 2:**
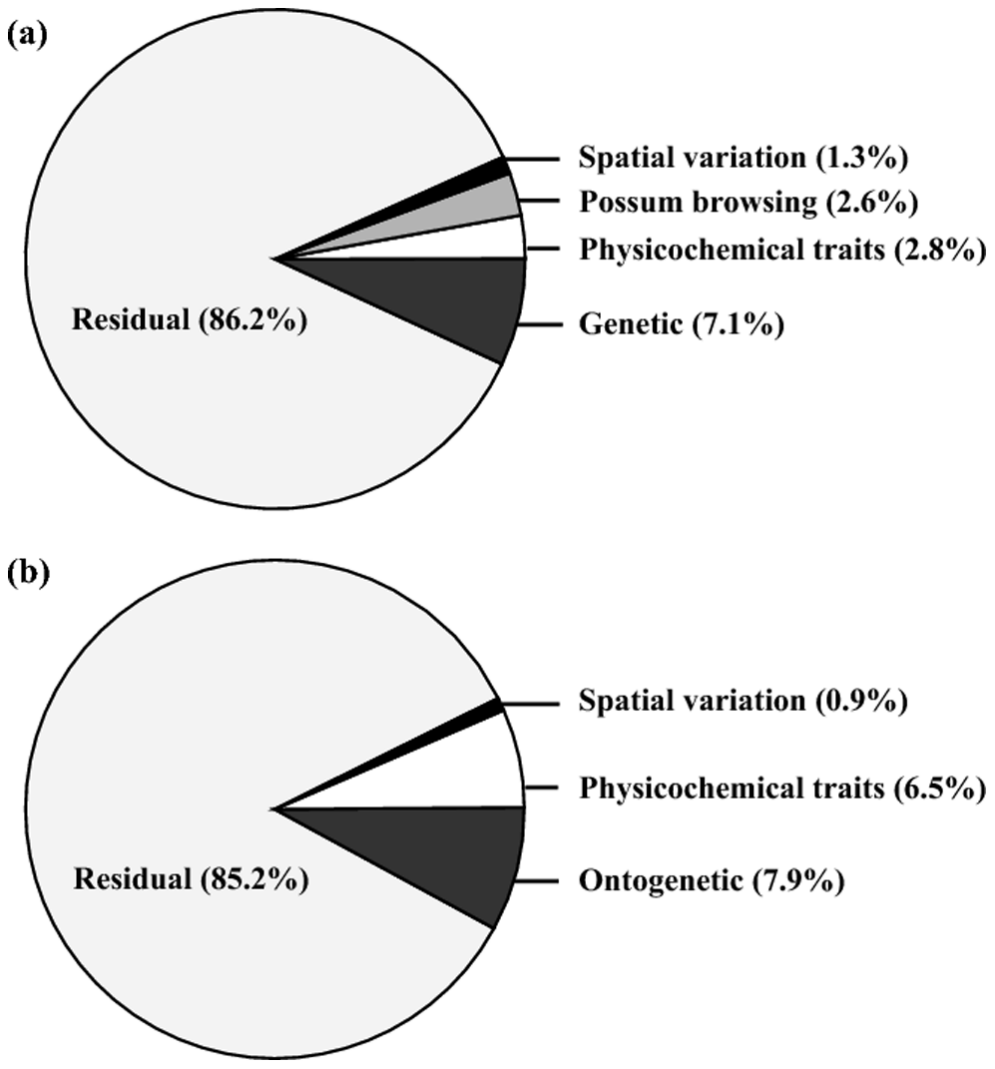
Pie charts summarizing the partition of variation in the dependent community Bray-Curtis dissimilarity matrices for (a) populations and (b) foliage types using Permanova+. The genetic effect represents only the variation between populations, as the family within population variation was not significant.

Two other factors could also influence variation in the arthropod and fungal community: NIR spectra variation (physicochemical properties) and variation in possum browsing. We tested whether population alone accounted for variation in the community after accounting for these two factors. By comparing the reduction in the percent variation attributed to population differences before and after including the NIR spectra principal components as covariates, we showed physicochemical properties of leaves accounted for 18% of the differences in community composition between populations. However, there were still significant differences in dependent community composition between populations (PERMANOVA model III, Pseudo-F_1,221_ = 4.3, p<0.001). On juvenile leaves, 5.7% of the variation in dependent community composition was explained by physicochemical properties of leaves, population 7.8% and spatial variation (ie. replicate) within the trial 1.3%. *Trichosurus vulpecula* browsing also significantly influenced variation in dependent community composition (PERMANOVA model V, Pseudo-F_4,221_ = 7.6, p<0.001) when accounted for first in the analyses. By comparing the reduction in the percent variation attributed to physicochemical properties of leaves and population differences before and after including *T. vulpecula* browsing as a covariate, it was shown that *T. vulpecula* browsing removed 44% of the variation in physicochemical properties (NIR spectra principal components) and 9.5% of the differences in community between populations. The browsing effect was still significant (PERMANOVA model VI, Pseudo-F_4,221_ = 1.8, p = 0.018) when placed after the principal components derived from the NIR spectra of juvenile foliage, indicating physicochemical properties do not account fully for the effect *T. vulpecula* browsing on community differences between populations. However, significance was lost (PERMANOVA model VII, Pseudo-F_4,221_ = 1.0, p = 0.455) when browsing was accounted for last in the analysis, indicating that the community effect of *T. vulpecula* browsing can be explained by a combination of population and physicochemical differences. The genetic effect (population) remained highly significant in each model. Overall, *T. vulpecula* browsing explained 2.6% of the variation in dependent community composition, physicochemical properties of leaves 2.8%, population 7.1% and spatial variation within the trial 1.3% (Fig. 2a– model V ).

**Figure 3:**
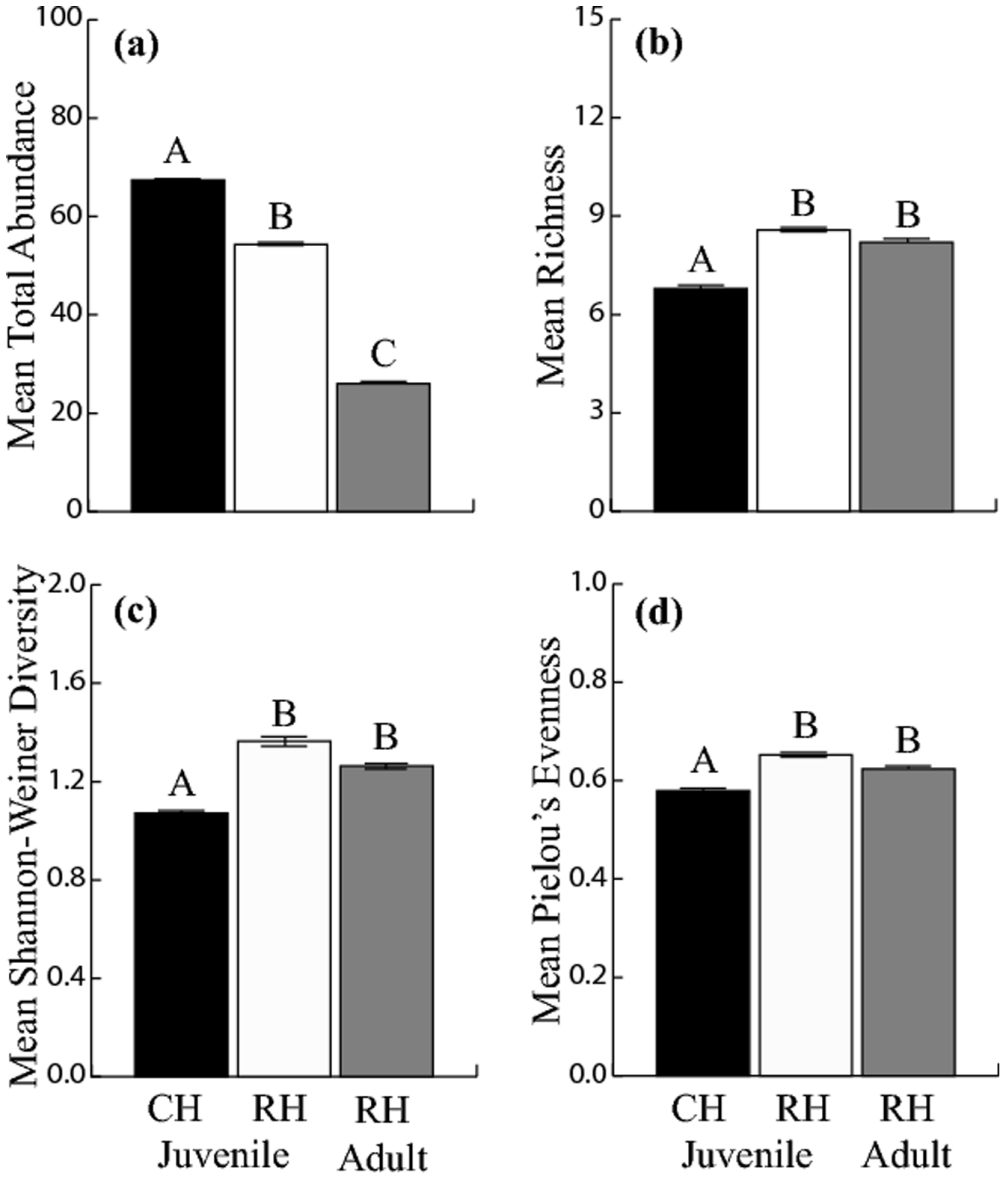
Mean community parameters for populations (CH and RH) and foliage types (juvenile and adult of RH) of *E. morrisbyi*. (a) Total abundance of all identified organisms (abundance), (b) the number of identified organisms (richness), (c) Shannon-Weiner diversity index, and (d) Pielou's evenness. Different letters above the bars represent significant differences between groups based on a linear mixed model for population differences and paired sample t-tests for foliage type differences. Error bars represent 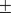 1 SE. All community parameters were calculated from the weighted abundance scores. Differences in community parameters between CH juvenile and RH adult foliage were not tested.

When we examined individual community parameters as opposed to overall community differences, we also found significant differences between populations. All community parameters investigated (richness, abundance, Shannon-Weiner diversity index, and Pieolou's evenness) were found to be significantly different between populations based on juvenile leaf samples after a Bonferroni adjustment to p = 0.00125 (Table 2). On average, more symptoms (richness) were found on trees from the RH population (Figure 3), while more overall damage (abundance) was found on trees from the CH population (Figure 3). Overall species diversity and evenness was greater on the juvenile foliage of the RH than CH population (Figure 3).

**Table 2:** Tests for the differences in community parameters between populations, families within populations and foliage types of *E. morrisbyi*.

	Juvenile Foliage				Juvenile and Adult Foliage	
	Population (Fixed)		Family(Pop.) (Random)		Foliage Type	
Community Parameter	Wald-F	P	χ2	P	t	P
Abundance (log10)	**18.3**	**<0.001**	0	1	−0.9	0.393
Richness	**27.4**	**<0.001**	0.4	0.53	**−12.7**	**<0.001**
Shannon-Weiner diversity	**76.9**	**<0.001**	0	1	−1.8	0.081

Statistical significance in community parameters between populations and family within populations was tested with generalized linear mixed model (model I). Replicate was not significant for any community parameter. Statistical significance in community parameters between foliage types was tested with paired sample t-tests. Foliage type t-values represent the mean difference in adult foliage minus juvenile foliage (A–J). Transformations, if necessary, are given in parentheses. Significant values after Bonferroni adjustment of p-values are in boldface.

**Figure 4:**
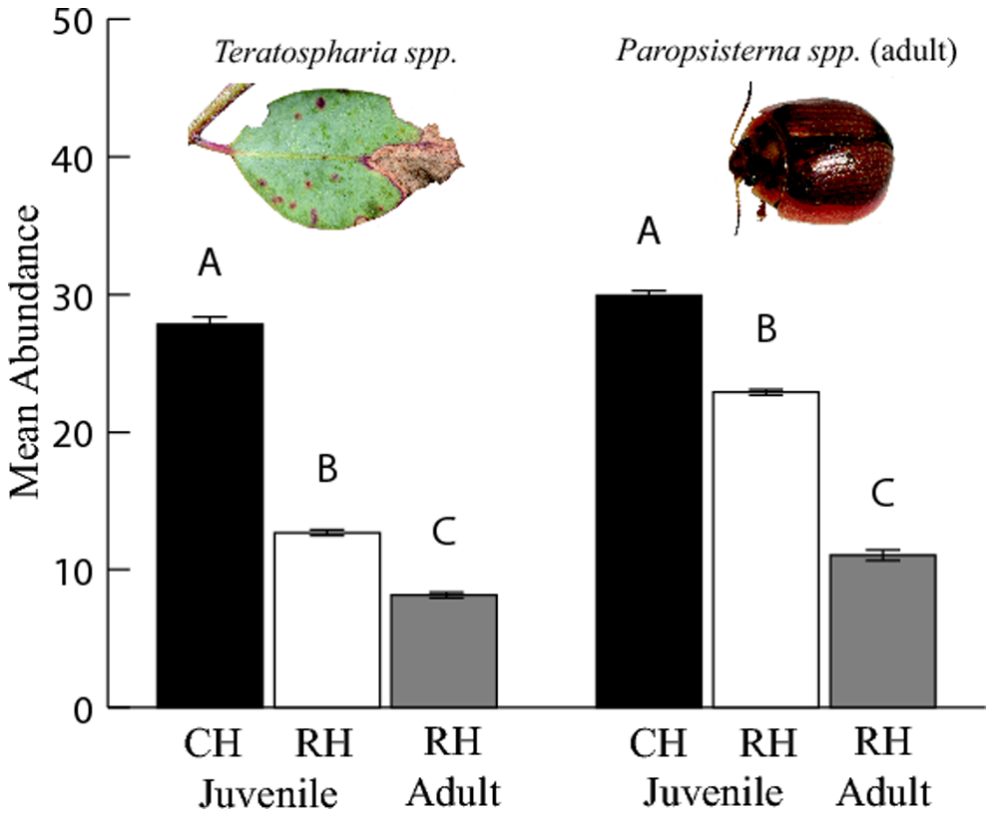
Mean abundance per sample of the two main symptoms driving community differences (fungal pathogen *Teratosphaeria spp.* and the leaf beetle *Paropsisterna spp.*) between populations (CH and RH) and foliage types (juvenile and adult of RH) of *E. morrisbyi*. Different letters above the bars represent significance between groups based on Kruskal-Wallis tests for population differences and paired sample t-tests for foliage type differences on the standardized abundance scores. Variation in causal organism abundances was not tested between CH juvenile and RH adult foliage. Error bars represent 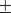 1 SE. Photographs of organisms taken by L. Forster.

Of the 60 symptoms identified on juvenile foliage forty-seven of these were found to be common between populations, but only twenty-one were found to occur on over ten percent of the juvenile foliage samples. A total of eight of the twenty-one symptoms found on more than ten percent of the samples were significantly different between populations after a Bonferroni adjustment of significance to p = 0.002 (Table 3), based on the twenty-one symptoms analyzed. Stepwise exclusions of these eight symptoms from the PERMANOVA community analysis (model V) showed only two symptoms of the original sixty were responsible for the variation in community composition detected between the juvenile foliage of the CH and RH populations. These highly influential symptoms were the fungal pathogen *Teratosphaeria spp.* and adult leaf beetle *Paropsisterna spp.*, which both had a greater abundance on the CH population (Figure 4).

**Table 3:** Significance of arthropod and fungal causal organisms found responsible for the variation in dependent community composition between populations and foliage types of *E. morrisbyi*.

	Juvenile Foliage		Juvenile and Adult Foliage	
	Population		Foliage Type	
Putative Causal Organism	Kruskal (χ2)	P	T	P
*Teratospharia spp.* (sqrt)	**68.6**	**<0.001**	**−5.2**	**<0.001**
*Paropsisterna spp*., adult (sqrt)	**13.8**	**<0.001**	**−9.5**	**<0.001**
*Gonipterus scutellatus*, larvae (sqrt)	29.4	<0.001	**−4.7**	**<0.001**
*Pachysacca samuelii*	18.7	<0.001	**−5.5**	**<0.001**
*Hymenoptera spp*. 3 (sqrt)	11	<0.001	**−2.7**	**0.008**
*Acrocercops laciniella* (sqrt)	4.1	0.043	**−6.7**	**<0.001**
*Ctenarytaina eucalypti*	2.8	0.095	**−5.5**	**<0.001**
*Hyalinaspis spp*. (sqrt)	0.6	0.428	**−3.3**	**0.002**

Organisms listed were those found to be the main symptoms influencing variation in community composition between populations and foliage types based on their stepwise elimination from the Permanova+ analysis. Causal organisms found to significantly influence the variation in dependent community composition between populations and foliage types from this step-down analysis are presented in boldface. Univariate tests of the significance in organism abundance are shown. Between populations this was tested with nonparametric Kruskal-Wallis tests. Statistical significance in organism abundance between foliage types was tested with paired sample t-tests. Foliage type t-values represent the mean difference in adult foliage minus juvenile foliage (Adult-Juvenile). Transformations, if necessary, are listed.

### Ontogenetic Variation in Foliar Organism Responses

A total of 60 symptoms of dependent arthropod and fungal species were identified on the RH population samples having both juvenile and adult foliage. There were highly significant differences in dependent communities on juvenile and adult leaves of the RH population (PERMANOVA model II, Pseudo-F_1,157_ = 12.92, p<0.001). This difference is clearly seen in the ordination space derived from the CAP analysis (Figure 1). Spatial variation in the trial was also found to be a significant factor in community composition for this model (PERMANOVA, Replicate: Pseudo- F_5,157_ = 1.25, p = 0.029). However, the variance components obtained from the PERMANOVA analysis showed that this influence was small (0.9% of total variation) when compared with the foliage type effect (13%).

The addition of principal components derived from the NIR spectra as covariates in the community PERMANOVA analysis of the ontogenetic samples showed seven of the principal components significantly influenced the dependent community composition. By comparing the reduction in the percent variation attributed to foliage type differences before and after including the NIR spectra principal components as covariates, it was shown they account for 39% of the differences in community composition explained by foliage types. However, even then the foliage type effect remained significant (PERMANOVA model IV, Pseudo-F_1,157_ = 2.8, p<0.001). Over all samples, the total influence of physicochemical properties of leaves explained 7.5%, ontogenetic variation 7.9% and spatial variation (ie. replicate) in the trial an insignificant 0.9% of the variation in dependent community composition (Figure 2b).

Of the community parameters investigated, only abundance was found to be significantly different between foliage types after a Bonferroni adjustment of significance to p = 0.0125 (Table 2). Abundance of symptoms was greatest on juvenile foliage (Figure 3). Of the 60 symptoms identified, forty-four of these were common between foliage types, eight were only found on the adult foliage and eight were only found on the juvenile foliage. Similar to the between populations data set, the majority of symptoms were rare and found on less than ten percent of the leaves. Of the twenty-one symptoms at higher frequencies, eight were significantly different between foliage types with a Bonferroni adjustment of significance to p = 0.002 (Table 3). Stepwise exclusions of these eight symptoms from the PERMANOVA community analysis (model IV) showed seven of the original sixty assessed symptoms were responsible for the variation in community composition detected between adult and juvenile foliage types of the RH population (Table 3). All seven symptoms responsible for the variation in community composition detected between foliage types were greater on the juvenile foliage of the RH population (only *Teratosphaeria spp.* and *Paropsisterna spp.* presented in Figure 4). An additional analysis of covariance (ANCOVA) showed that the sampling intensity of adult and juvenile foliage (ie. the number of leaves assessed) significantly influenced only four of the symptoms, however, this did not affect the results between foliage types.

## Discussion

Our study shows that both genetic (9.5%) and ontogenetic (13.0%) variation in *E. morrisbyi* significantly influences the composition of arthropod and fungal communities in this rare heteroblastic eucalypt. Both of these factors remained significant even after accounting for physicochemical properties (for both genetic and ontogenetic) and possum browsing (genetic only). The variation explained by genetic effects in this study was solely driven by differences between populations rather than families. Only four plant systems have quantified the size of genetic-based variation on dependent communities [2], [36]–[40]. Three of these systems used clonal replicates for their experiments, all of which exhibited high estimates of genetic influence across the systems (*Populus*–32–93%; *Oenothera*–30%; *Quercus–*7–26%). The other system studied the genetic variation found in a natural forest patch and showed less genetic influence (*Brosimum*–3.7–4.8%). The genetic effects found in our study were smaller than those found in other systems, but were still statistically significant. Our low genetic effect compared to other studies may be in part due to the scoring techniques as the other studies have assessed live organisms as opposed to the symptom-based approach used in this study. As mentioned in the methods, the possibility that symptoms may represent multiple organisms cannot be dismissed, which would potentially reduce host genotype specificity in our study.

There are many differences between studies quantifying host genetic effects on dependent communities. Nevertheless, the low effect values observed in our system may relate to the *Brosimum* system in unexpected ways, despite *Brosmium* being evergreen trees located in warmer tropical climates. The high diversity of trees found in tropical regions [41] along with the moderately high plant diversity and numerous *Eucalyptus* species co-occurring in sclerophyll forests [42] may reflect why the influence of genetic variation on community composition was lower in the *Brosimum* and current study, respectively. A study of insect herbivores in a tropical lowland forest in New Guinea has shown that host specificity decreases with increasing number of tree species in a forest [43]. While this has not been studied in the *Eucalyptus* system, the lower relative influence of genetic variation found in this study may be reflecting a greater frequency of generalist herbivores compared to the other systems. This concept may be further supported by the study species being rare, with common garden trials outside of their natural populations. This distance from natural stands would reduce the occurrence of possible specialist herbivores associated with the species.

With regard to the organisms in the community, we found only two, the fungal pathogen *Teratosphaeria spp.* and the leaf beetle *Paropsisterna spp.*, were driving the differences between populations. Genetic variation in the susceptibility to both *Teratosphaeria spp.* [16], [44] and *Paropsisterna spp.* [17], [45] have been well studied in the other *Eucalyptus* systems, with the presence of these organisms having a deleterious effect on plant growth [44], [46]. In contrast, seven organisms were driving community differences between foliage types. The proportion of organisms driving the genetic-based community differences (3–13%) in this study is in contrast to other systems that have found a greater proportion (45%–100%) of community organisms as significantly different between genotypes [26], [47]. This could be due to differences in the number of organisms assessed between the studies, with the studies showing greater proportions of significance assessing less than twenty organisms compared to the sixty symptoms identified in this study. As previously discussed, this may also be due to differences in the frequency of generalist and specialist comprising the dependent communities, as one study has shown increased herbivore diversity to be associated with additional generalists [48]. This may also be reflected in recordings of higher insect herbivory in eucalypts compared to temperate communities of the northern hemisphere [49], which may also explain the response of organisms to ontogenetic versus genetic differences, where a generalist herbivore may respond to the more readily discernable morphological and chemical differences between foliage types than the more subtle differences between populations.

Investigation of the mechanisms driving the variation between communities found that previous *T. vulpecula* browsing accounted for 10% of the genetic effect. This could be due to diffuse interactions [50] by which preferences of insects is in part affected by *T. vulpecula* browsing. This interaction may be due to direct or indirect effects. Direct effects include direct ecological interference such as *T. vulpecula* eating preferred foliage of certain organisms feeding on the plants at the same time (e.g. competition). Indirect effects could include: (1) browsing causing the retention of juvenile foliage, influencing the abundance and richness of organisms; (2) induced changes in plant chemistry and/or morphology altering the habitat for arthropod and fungal colonization. To date, however, few studies have demonstrated induction of chemical defenses in eucalypts [51], [52], and so other factors may be at play.

We found the variation in physicochemical traits accounted for 15% of the genetic and 37% of the ontogenetic effects explaining community differences. Numerous studies have shown the importance of variation in traits, such as foliar chemistry, as mechanisms influencing herbivory [53], [54]. While both *T. vulpecula* browsing and physicochemical traits did explain some of the population effect, there was still a clear genetic effect (7.1%) influencing community variation. This genetic variation may be affecting the community through: (1) traits undetected in NIR spectra; (2) variation in tree architecture [55]; (3) plant growth [40]; (4) other diffuse interactions, perhaps from a third trophic level including predatory birds and insects [56].

The influence of genetic-based effects on community composition in this study shows the importance of maintaining the two populations of this species as distinct management units. For example, the choice of just one of these populations for conservation would lead to a different biotic community trajectory compared to the choice of the other population. Such different trajectories would depend on the stability of genetic-based differences across life history stages [57], and in contrasting environments. Understanding extended genetic effects in species of conservation concern allows us to examine the evolutionary relationships between the host plant and their herbivores, to better understand the developing community trajectory in newly planted restored forests and to provide insight for managers on how these rare species may be best managed for the future [3], [58].

## Supporting Information

Figure S1 **Photographs of select causal organism symptoms on** ***E. morrisbyi*** **foliage.** (a) *Acrocercops laciniella*, (b) *Aulographina eucalypti*, (c) *Diphucephala colaspidoides*, (d) *Eurymeloides bicincta* (eggs), (e) *Gonipterus scuttelatus* (larvae), (f) *Paropsisterna spp.*, (g) *Paropsisterna agricola* (larvae), (h) *Sonderhenia eucalyptorum*, (i) *Teratosphaera spp*., and (j) *Uraba lugens*. Red circles highlight damage types.
(PDF)

Table S1 **Organism identifications and symptom descriptions.**
(DOCX)
