# Supplementary material for: Genetic and Ontogenetic Variation in an Endangered Tree Structures Dependent Arthropod and Fungal Communities
Source: PLoS One. 2014 Dec 3;9(12):e114132. doi: 10.1371/journal.pone.0114132 (PMC4254790; doi:10.1371/journal.pone.0114132)
Supplement: Figure S1 — Photographs of select causal organism symptoms on E. morrisbyi foliage. (a) Acrocercops laciniella, (b) Aulographina eucalypti, (c) Diphucephala colaspidoides, (d) Eurymeloides bicincta (eggs), (e) Gonipterus scuttelatus (larvae), (f) Paropsisterna spp., (g) Paropsisterna agricola (larvae), (h) Sonderhenia eucalyptorum, (i) Teratosphaera spp., and (j) Uraba lugens. Red circles highlight damage types. (PDF) [file pone.0114132.s001.pdf]

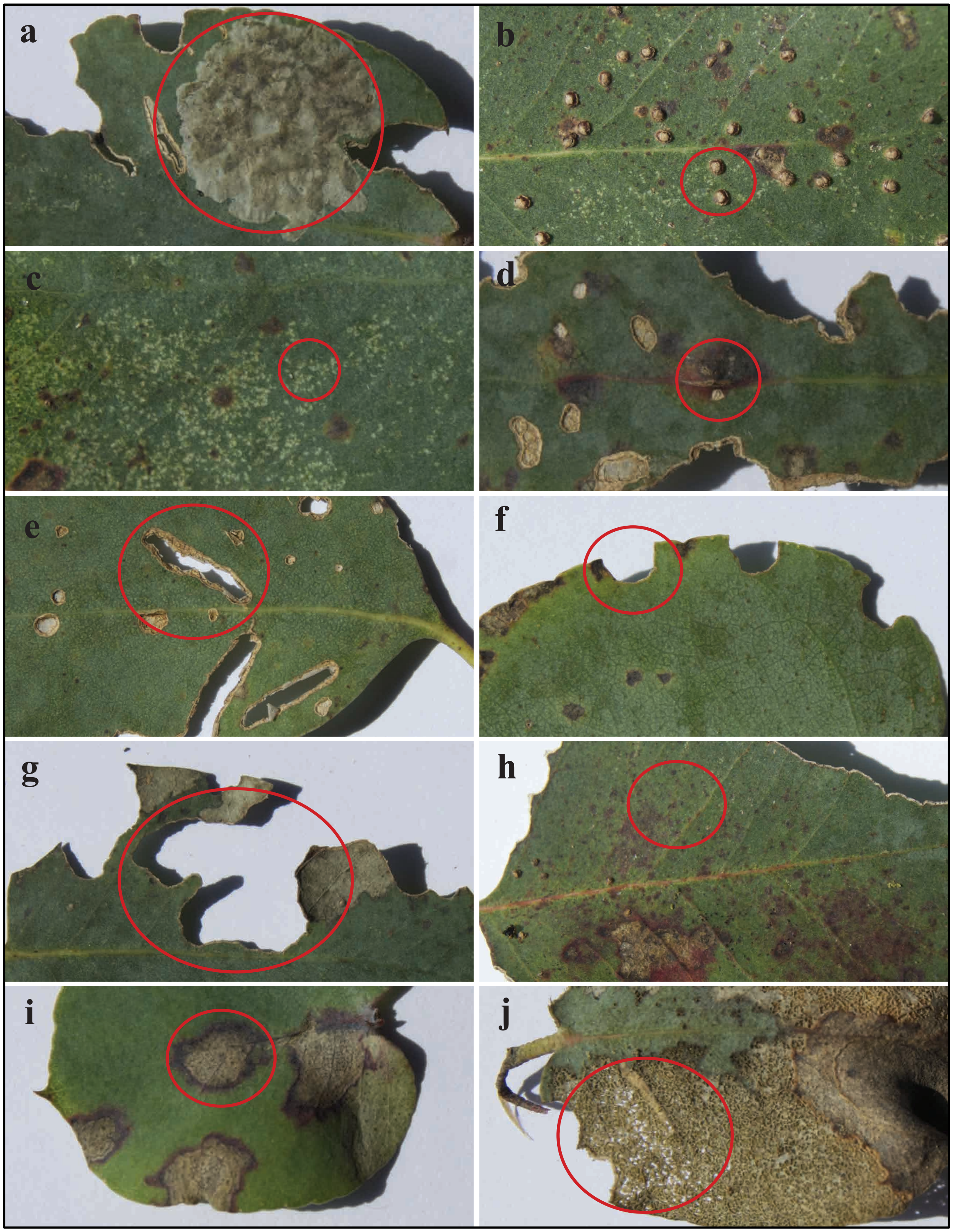

Figure S1. Photographs of select causal organism symptoms on *E. morrisbyi* foliage (See Table S1 for descriptions). (a) *Acrocercops laciniella*, (b) *Aulographina eucalypti*, (c) *Diphucephala colaspidoides*, (d) *Eurymeloides bicincta* (eggs), (e) *Gonipterus scuttelatus* (larvae), (f) *Paropsisterna spp.*, (g) *Paropsisterna agricola* (larvae), (h) *Sonderhenia eucalyptorum*, (i) *Teratosphaera spp.*, and (j*) Uraba lugens*. Red circles highlight damage types.
