# Supplementary material for: Genetic and Ontogenetic Variation in an Endangered Tree Structures Dependent Arthropod and Fungal Communities
Source: PLoS One. 2014 Dec 3;9(12):e114132. doi: 10.1371/journal.pone.0114132 (PMC4254790; doi:10.1371/journal.pone.0114132)
Supplement: Table S1 — Organism identifications and symptom descriptions. (DOCX) [file pone.0114132.s002.docx]

**Table S1.** Organism identifications and symptom descriptions

| **Organism causing leaf damage** | **Order** | **Description of Leaf Damage** |
| --- | --- | --- |
| *Diphucephala colaspidoides* | Coleoptera | finely rasped epidermal cells |
| *Gonipterus scutellatus species complex* - failed feeding by larvae | Coleoptera | bright green rectangle of the size and shape of larval Gonipterus tracks but with the epidermis still intact |
| *Gonipterus scutellatus species complex* - feeding by larvae | Coleoptera | parallel-sided slots though the leaf, or with a transparent layer of cuticle on the other surface of the leaf remaining; the slot-like tracks vary in length and density; additionally a pair of straight, brown, sclerotised lines extending from a slot up to 5mm across the lamina. |
| *Paropsisterna agricola* larva | Coleoptera | chewed area narrower at leaf margin then expanding irregularly |
| *Paropsisterna* sp. adult | Coleoptera | leaf margins appear 'scalloped' |
| *Cecidomyiidae* sp. 1 | Diptera | pyramid-shaped galls on one surface of lamina connected to a more rounded protusion on the other surface; multiple but separate (average height 8mm) |
| *Cecidomyiidae* sp. 2 | Diptera | single, more rounded pyramid-shaped gall on one surface of lamina connected to a rounded protrusion on the other surface; (average height 8mm) |
| *Diptera* sp. 2 | Diptera | tiny, brown scattered lesions at leaf apex, not prominent |
| *Diptera* sp. 3 | Diptera | tiny, brown lesions along leaf margin, in an indistinct row |
| *Diptera* sp. 4 | Diptera | tiny, brown, scaly lesions along leaf margin, in two indistinct rows |
| *Diptera* sp. 5 | Diptera | reddish, spherical galls (2mm), along midvien at apex of leaf |
| *Fergusonina* sp. 1 | Diptera | large (to 10mm diameter), irregular, fleshy gall without distinct structure, along leaf margin |
| *Hymenoptera* sp. 5 | Diptera | tiny, pointy, pimple-like lesion scatteres on leaf |
| *Aulographina eucalypti* | Fungal | concentric, necrotic rings visible on only one side of lamina which may be purple-edged in winter; ascospores 2 cells |
| *Cryptosporiosis eucalypti* | Fungal | irreguar, warty, reddish-brown, lesions |
| *Fumago vagans* | Fungal | black, sooty, fungus; secondary to damage from sap feeding insects. |
| *Pachysacca samuelii* | Fungal | connective tissue finely warty, not sclerotic, frequently with light margins |
| *Sonderhenia eucalyptorum* | Fungal | small purple spots, not, or hardly, elevated that become necrotic with age |
| *Teratosphaeria* spp. | Fungal | small to large irregular blotches which commonly coalesce; purple, red-brown and gray; visible on both surfaces of the lamina |
| *Eurymelinae* sp. 1 | Hemiptera | small, reddish, linear slerotism of vein adjacent to midvein |
| *Eurymelinae* sp. 2 | Hemiptera | small, reddish, sclerotic spots on veins (pierced for sap feeding) |
| *Eurymelinae* sp. 3 | Hemiptera | small, reddish, sclerotic spots between veins (pierced for sap feeding) |
| *Eurymelinae* sp. 4 | Hemiptera | small, reddish, sclerotic spots adjacent to mid vein on abaxial surface |
| *Eurymeloides bicincta* | Hemiptera | small, reddish, sclerotic spots along mid vein on abaxial surface |
| *Eurymeloides bicincta* eggs | Hemiptera | small, reddish, linear slerotism along midvein |
| *Australopsylla* sp. | Homoptera: Psyllidae | small (2mm) wrinkled spherical galls on adaxial surface with slight pit on abaxial surface containing a flat, white lerp |
| *Creiis* sp. 1 | Homoptera: Psyllidae | transparent, leongate lerp, abaxial surface of lamina |
| *Ctenarytaina eucalypti* | Homoptera: Psyllidae | white,waxy secretion, reddening around vein pierced for feeding |
| *Glycaspis* sp. 1 | Homoptera: Psyllidae | white domed lerp covered with strands of waxy secretions; sometimes black strands due to sooty mould |
| *Hyalinaspis* sp. 1 | Homoptera: Psyllidae | transparent, clamshell-like lerp (3mm), with psyllid covered in loose, white strands |
| *Hyalinaspis* sp. 2 | Homoptera: Psyllidae | psyllid with translucent, brown, clamshell-like lerp (average 5mm) |
| *Hyalinaspis* sp. 3 | Homoptera: Psyllidae | Psyllid with yellow clamshell-like lerp (average 8 mm) |
| *Hyalinaspis* sp. 4 | Homoptera: Psyllidae | Psyllid with white clamshell-like lerp containing 2 halves (average 5mm) |
| *Hyalinaspis subfasciata* | Homoptera: Psyllidae | psyllid with transparent clamshell-like lerp (average 9mm) |
| *Lasiopsylla rotundipennis* | Homoptera: Psyllidae | almost flat, white lerps, often in groups, large, approx 3-5mm |
| *Schedotrioza multitudinea* | Homoptera: Psyllidae | small (to 5mm diameter), irregular, fleshy, spherical galls; often joined making shape indistinct |
| *Schedotrioza* sp. 1 | Homoptera: Psyllidae | mostly oval-shaped hole with brown margin of exposed mesophyll tissue (up to 4mm), regular distance from leaf margin |
| *Schedotrioza* sp. 3 | Homoptera: Psyllidae | slightly elliptical gall on abaxial midvein of leaf; approx 3mm |
| *Schedotrioza* sp. 4 | Homoptera: Psyllidae | tiny gall (approx 1.5mm) at base of leaf, on midvein, frequently with erupted opening |
| *Schedotrioza* sp. 5 | Homoptera: Psyllidae | tiny gall(1mm) near abaxial margin of leaf, no syptom on adaxial surface |
| *Schedotrioza* sp. 6 | Homoptera: Psyllidae | gall causing lamina to bulge on one side leaving a small pit on the other side |
| *Hymenoptera* sp 2 | Hymenoptera | straight line of pimple-like lesions along margin of leaf |
| *Hymenoptera* sp. 6 | Hymenoptera | tiny pimple-like lesions forming a ring approx 4mm diameter |
| *Hymenoptera* sp. 7 | Hymenoptera | circular gall with irregular surface, some sclerotisation; not visible on other surface of lamina |
| *Hymenoptera* sp. 8 | Hymenoptera | separate but numerous sclerotic, pimple-like galls across lamina (< 1mm diameter), |
| *Ophelimus eucalypti* | Hymenoptera | spherical galls 3-5mm embedded in leaf, prodruding and visible from both surfaces; often with a purplish hue |
| *Phylacteophaga froggatti* | Hymenoptera: Pergidae | broad, flat, transparent blister of epidermal cells, frequently covering the entire abaxial lamina |
| *Acrocercops laciniella* | Lepidoptera | an irregular, transparent trail or, in later stages, blister of epidermal cells remaining after 'leaf mining' by the larvae which consumes mesophyll tissue |
| *Agriophara gravis* | Lepidoptera | a dead leaf joined by web to a live one to provides a shelter for the caterpillar which grazes on surface tissue |
| *Heliozela prodela* | Lepidoptera | shot hole with smooth edges up to 4mm |
| *Heliozela* sp. 2 | Lepidoptera | brown rectangular blister on adaxial lamina with an exit hole at one end on the abaxial surface |
| *Hemibela heliotricha* | Lepidoptera | 5-10mm twig upright on mid-vein, and standing perpendicular to lamina. Twig hollow to provide shelter for a larva |
| *Hyalarcta* sp. 1 | Lepidoptera | small (up to 5mm) brown patches of epidermis deeply grazed between veins but not penetrating the other laminal surface |
| *Hypertropha tortriciformis* | Lepidoptera | entire margin of leaf rolled and loosely webbed together; veins around which feeding occurs become quite dark |
| *Mnesampela privata* | Lepidoptera | leaves at tips of branches loosely webbed together; tracks of grazed parenchyma become dark brown |
| *Oecophoridae* sp. 1 | Lepidoptera | three live leaves strongly held closely together with silk |
| *Tortricidae sp.* 1 | Lepidoptera | margin of leaf rolled and held with parrallell strands of silk |
| *Uraba lugens* | Lepidoptera | skeletonised i.e. only veins are visible following grazing of parenchyma |
| Unknown 1 |  | regular inverse-scalloping along leaf margin |
| Unknown 2 |  | herbivory of leaf margin seems to have occurred when the leaf was young and expanding, resulting in distortion so it is difficult to assign a specific organism to the cause |
| Unknown 3 |  | tear-shaped holes adjacent to midvein; appox 5mm long |
| Unknown 4 |  | brown lesion forming a straight line extending up to 10mm |
| Unknown 6 |  | similar straight line lesion as unknown 4, but parallel pairs of lesions approx. 3mm apart |
